# Frequency of breaks, amount of muscular rest, and sustained muscle activity related to neck pain in a pooled dataset

**DOI:** 10.1371/journal.pone.0297859

**Published:** 2024-06-25

**Authors:** Markus Koch, Mikael Forsman, Henrik Enquist, Henrik Baare Olsen, Karen Søgaard, Gisela Sjøgaard, Tove Østensvik, Petter Nilsen, Lars Louis Andersen, Markus Due Jacobsen, Mikkel Brandt, Rolf Westgaard, Paul Jarle Mork, Xuelong Fan, Morten Wærsted, Kaj Bo Veiersted

**Affiliations:** 1 National Institute of Occupational Health, Research Group for Work Psychology and Physiology, Oslo, Norway; 2 Division of Occupational and Environmental Medicine, Department of Public Health Sciences, Karolinska Institute, Stockholm, Sweden; 3 Division of Occupational and Environmental Medicine, Lund University, Lund, Sweden; 4 Department of Sports Science and Clinical Biomechanics, University of Southern Denmark, Odense, Denmark; 5 Department of Clinical Research, University of Southern Denmark, Odense, Denmark; 6 Norwegian Institute of Bioeconomy Research, Ås, Norway; 7 National Research Centre for the Working Environment, Musculoskeletal Disorders and Physical Workload, Copenhagen, Denmark; 8 Department of Industrial Economics and Technology Management, Norwegian University of Science and Technology, Trondheim, Norway; 9 Department of Public Health and Nursing, Norwegian University of Science and Technology, Trondheim, Norway; Universidade Federal de Sao Carlos, BRAZIL

## Abstract

**Background:**

Neck pain remains a persistent challenge in modern society and is frequently encountered across a wide range of occupations, particularly those involving repetitive and monotonous tasks. It might be expected that patterns of trapezius muscle activity at work, characterized by few breaks and prolonged periods of sustained muscle activity, are linked to neck pain. However, previous cross-sectional studies have generally failed to establish a definitive association. While some longitudinal studies have suggested that extended periods of heightened muscle activity could be a risk factor for neck pain, these findings often relied on limited participant numbers or specific professional groups. This study aimed to investigate the relationship between trapezius muscle activity and neck pain by pooling data from seven Scandinavian research institutes encompassing a diverse range of occupational backgrounds.

**Methods:**

Electromyographic (EMG) data for the upper trapezius muscle, collected during working hours, were coupled with questionnaire responses pertaining to neck pain, individual characteristics, and potential confounding variables for a total of 731 subjects. Additionally, longitudinal data from 258 subjects were available. The various EMG datasets were consolidated into a standardized format, and efforts were made to harmonize inquiries about neck pain. Regression analyses, adjusting for sex and height, were conducted to explore the associations between muscle activity variables and neck pain. An exposure index was devised to quantify the cumulative neck load experienced during working hours and to differentiate between various occupational categories.

**Results:**

The cross-sectional data displayed a distinct pattern characterized by positive associations for brief periods of sustained muscle activity (SUMA) and negative associations for prolonged SUMA-periods and neck pain. The longitudinal data exhibited a contrasting trend, although it was not as pronounced as the cross-sectional findings. When employing the exposure index, notable differences in cumulative muscle load emerged among occupational groups, and positive associations with longitudinal neck pain were identified.

**Discussion:**

The results suggest that individuals with neck pain experience higher cumulative workloads and extended periods of muscle activity over the long term. In the short term, they appear to compensate by taking frequent short breaks, resulting in a lower cumulative workload. Regardless of their occupation, it is crucial to distribute work breaks throughout the workday to ensure that the cumulative load remains manageable.

## Introduction

Despite decades of research, neck pain continues to impose a significant economic burden, encompassing the costs associated with its treatment, productivity losses, and premature retirement [1]. On a global scale, neck pain ranks as the fourth most prevalent cause of years lived with reduced health [2]. From 1990 to the present day, neck pain has emerged as a progressively more substantial issue when juxtaposed with other health concerns [3] and has witnessed a roughly 20% increase in prevalence between 1990 and 2017 [4].

For numerous years, researchers have delved into potential mechanisms underlying the onset of neck pain, yet a consensus on likely explanatory factors remains elusive [5]. These factors encompass acute muscle and support tissue overloads in the neck [6], prolonged exposure to static conditions [5, 7, 8], or a combination thereof, often accompanied by additional exposures that culminate in structural and functional alterations within the central nervous system [9]. It is plausible that multiple mechanisms coexist, with varying mechanisms coming into play depending on whether the outcome is acute or chronic neck pain.

The role of muscular load during work as a potential risk factor for neck pain has been investigated extensively in the past, with surface electromyography (EMG) often employed to monitor the amplitude of the upper trapezius muscle [10–12]. Both mechanical factors, such as manual handling and maintaining static postures, and psychological factors, like high job demands and monotonous tasks, can be conceptually linked to increased activation of the neck muscles [10–12]. Persistent tension in the neck muscles, leading to a lack of muscular rest, may disrupt muscle homeostasis, affect tendon metabolism, and influence the release of analgesic or pain-sensitizing substances [13].

Despite conflicting findings from previous studies, it is generally observed that heightened activity in the trapezius muscle does not seem to be directly associated with concurrent pain [5, 14–16]. Nevertheless, there are several findings indicating that individuals experiencing neck pain exhibit increased muscle tension compared to those without pain due to what is termed ‘stress exposure’ [17, 18]. This phenomenon has also been observed in a prospective study, where individuals exhibited increased muscle tension before the development of neck pain [19].

Conversely, an increase in muscular rest may serve as a preventive measure against neck pain. In most studies, “muscular rest” is defined as very low muscular activity, typically falling below 0.5% of the highest level achieved during maximal voluntary contraction of the trapezius muscle (MVE). Several cross-sectional studies examining workers using EMG on the neck muscles have reported associations between a low level of muscular rest and increased reports of neck pain [14, 20–24]. However, other studies have failed to establish such a connection [25–28]. Prospective studies have also been conducted in which muscle activity was initially measured during work, followed by the monitoring of pain development. The limited studies employing this design have shown that muscular rest reduces the risk of developing neck pain [29, 30].

Additionally, short interruptions in muscle activity during static and repetitive tasks, characterized as “EMG gaps” lasting from 0.2 to 2 s, have been demonstrated to be effective in preventing neck pain [29]. Therefore, there is substantial evidence suggesting that the overall duration of muscular rest plays a pivotal role in preventing neck pain. However, there remains a gap in our understanding regarding the precise amount of muscular rest required and the optimal distribution of activity and rest for prevention purposes.

An alternative approach involves scrutinizing muscle activation patterns to assess the associations between muscular load and neck pain. Previous studies have delineated these loads by segmenting them into periods of sustained muscular activity (SUMA), characterized by the absence of muscular rest and, thus, fewer opportunities for recovery. Originally labeled as sustained low-level muscle activity (SULMA), these periods were essentially defined as continuous activity exceeding 0.5% of maximal voluntary contraction of the trapezius muscle (MVE), lasting from more than 1.6 to over 5 s and extending up to greater than 20 min without muscle rest [31].

A study conducted among forest machine operators revealed an elevated risk of neck pain after one year for individuals who had experienced multiple periods exceeding 8 min with SUMA [31]. Similarly, a study involving hairdressers, electricians, and office workers early in their careers discovered an increased risk of reporting neck/shoulder pain for activity periods lasting more than 4 min [32] and, more specifically, a tripled risk if over half of their working time consisted of periods with sustained activity exceeding 4 min [33].

In summary, the findings regarding the associations between trapezius muscle activity and neck pain exhibit inconsistency and occasional conflicts. This variability could potentially be attributed to the fact that previous studies were predominantly conducted across diverse occupations and with smaller participant pools. In a preventive context, understanding the maximum tolerable muscular load during a typical workday and determining the appropriate frequency and duration of breaks based on specific job tasks is both relevant and essential.

### Aim

This study aims to investigate the relationship between trapezius muscle activity, including the frequency of breaks, duration of muscular rest, SUMA, and the occurrence of neck pain. In addition, an exposure index will be elaborated to grasp an overall exposure including activity and rest. We utilize a combined dataset from seven Scandinavian research institutes, which encompasses a significant number of workers from diverse occupational backgrounds.

### Hypotheses

We assume that a high median muscular activity during the working day, frequent high peak loads, sustained muscle activity, and higher values in the exposure index of the trapezius muscle are positively associated with neck pain.

On the opposite, we expect the number of micropauses and a high number and total time of periods with no/low muscle activity (“pauses”/“gaps”) being negatively associated with neck pain.

## Methods

For this study, we collected EMG data for the upper trapezius muscle during working hours and gathered related questionnaire data from 756 participants across seven research institutes. All of the data utilized in this study have been previously employed in other research studies [34–51]. At the National Institute of Occupational Health (STAMI), all of these datasets were amalgamated into a single, comprehensive dataset. The data underwent a rigorous three-step evaluation process.

First, we checked for data completeness, ensuring that both EMG data and questionnaire responses were available for each participant. Second, the EMG data was subjected to quality control measures, verified for format consistency, and transformed into a standardized format (details provided below). Third, the questionnaire data was also scrutinized for quality, and scales were compared, partly inverted, and matched where necessary (details below).

Following these quality control procedures and modifications, the final dataset comprised 731 participants. This dataset included EMG data for both the left (n = 547) and right (n = 726) trapezius muscles. Additionally, longitudinal questionnaire data on pain outcomes were available for 258 participants.

### Study population

All datasets included information on participants’ age, sex, weight, height, and profession answered by questionnaire. Based on the questions used in the various studies, some of them over 20 years old, we assume that the study participants responded in relation to their biological sex, whether they chose female or male. Moreover, data regarding dominant hand preference were available for 345 participants, with 59 individuals favoring their left hand and 286 their right hand. A comprehensive summary of the study population is provided in Table 1. Participants within the final dataset represented a diverse range of 32 different occupations. Additional details about the data sources contributing to the occupational groups included in the final dataset can be found in S1 Table.

**Table 1:** Study population.

Participants:	n = 731		
	**Mean (SD)**		
Age [years]	39.97 (12.03)		
Weight [kg]	76.22 (14.97)		
Height [cm]	174.02 (9.54)		
BMI [kg/m^2^]	26.31 (20.63)		
	**Sex**		** **
** Profession**	**Male**	**Female**	**Total**
Assembly worker	16	9	25
Assistant worker	3	0	3
Brewery worker	0	4	4
Bricklayer	22	0	22
Carpenter	17	0	17
Cleaner	1	11	12
Concrete worker	37	0	37
Cook or kitchen helper	4	4	8
Electrician	16	0	16
Engineer	2	1	3
Firefighter	2	0	2
Foreman	5	0	5
Gardener/forest worker	4	1	5
Hairdresser	0	36	36
Harvester / driver	92	2	94
Health care personal	6	70	76
Helicopter pilot	17	1	18
Machine operator	1	3	4
Meat cutter	29	6	35
Mechanic	4	0	4
Office worker / Secretary	28	79	107
Postal worker	35	1	36
Project manager/leader	8	5	13
Retail personal	13	32	45
Rubber mixing	1	7	8
Student	0	5	5
Surgeon	17	5	22
Warehouse worker	29	9	38
Windscreen inspection	3	7	10
Other occupations	11	3	14
Working with various tasks	2	5	7
**Total**	**425**	**306**	**731**

### Objective measures–EMG

As previously mentioned, this study utilized muscle activity data from the upper trapezius muscle during working hours, which was sourced from seven different research institutes.

The data from each institute had previously been used in publications and met specific quality standards for sensor placement, measurements, and data processing [52, 53]. An overview of the EMG measurement specifications for the various datasets can be found in the protocol paper for this study [54].

The data was provided in root-mean-square (RMS) format, with varying RMS lengths, and was normalized to the maximum voluntary effort (MVE). To ensure consistency, all the data was interpolated using spline interpolation to achieve a common frequency of 8 Hz.

The data underwent a comprehensive quality check, which included assessing data presence, overall data quality, the presence of artifacts, periods with missing data, normalization to MVE, and noise levels. A common noise correction procedure was applied to the pooled dataset, following the methodology used in prior studies. Specifically, the noise level was determined as the minimum of the moving average over 19 samples (equivalent to 2.375 s) and subtracted from the corresponding samples [14, 55, 56].

Any data that contained MVE measurements at the beginning or end of the measurement period was excluded from the analysis. Notably, there was no available information regarding planned breaks during the working day; consequently, all measurement data included muscle activity during work breaks, with the exception of 14 measurements from one dataset where breaks were deliberately excluded. Additionally, measurements with a duration of less than 60 min were excluded from the dataset to ensure data consistency and reliability.

For each participant, the following variables were calculated bilaterally: Relative resting time (RRT), as the total duration of muscle activity < 0.5% MVE related to the total measurement duration [57]; The number of gaps per minute with muscle activity < 0.5% MVE, was defined as the frequency of episodes of muscular rest with a minimal duration of 0.125 s [14] The 10^th^ percentile of muscle activity (static muscle activity level) [56]; the 50^th^ percentile of muscle activity (center or median of muscle activity) [56]; 90^th^ percentile (peak level of muscle activity during the total working shift) [56]; Various lengths of periods with SUMA > 0.5% MVE (interval range: 1.5–5 s to > 20 min) was also analyzed [31]. A SUMA was defined as a period with continuous muscle activity > 0.5% MVE with a duration > 1.5 seconds or more (interval range: 1.5–5 s to > 20 min), and numbers of these periods were analyzed per hour. The longer sample time for SUMA was chosen to avoid that very short gaps with questionable physiological effect interrupted the analyses of sustained activity.

An exposure index (EI), representing the cumulative exposure/rest distribution during the working day, was calculated by determining muscle activity into proportions (% of time) of three different activity levels for each period of 10 min (Muscular rest < 0.5% MVE [27, 29]; Low activity: 0.5–7% MVE; High activity: > 7% MVE [58, 59]) from the start to the end of the working day.

The EI was calculated as followed:

$$\begin{array}{c} E_{Index}T_{n}=-2*P_{Rest(Tn)}+P_{Low(Tn)}+2*P_{High(Tn)}\\ E_{Index\_cumulativ}T_{n}=E_{Index}T_{n}+E_{Index}T_{n-1},with\;E_{Index}T_{0}=0 \end{array}$$
Tn: Specific period (e. g. T_1_ = 0–10 min); P_Rest_ = Proportion of muscular rest; P_Low_ = Proportion of low muscular activity; P_High_ = Proportion of high muscular activity. Each proportion was calculated as percent of time during each 10min period.

**Fig 1:**
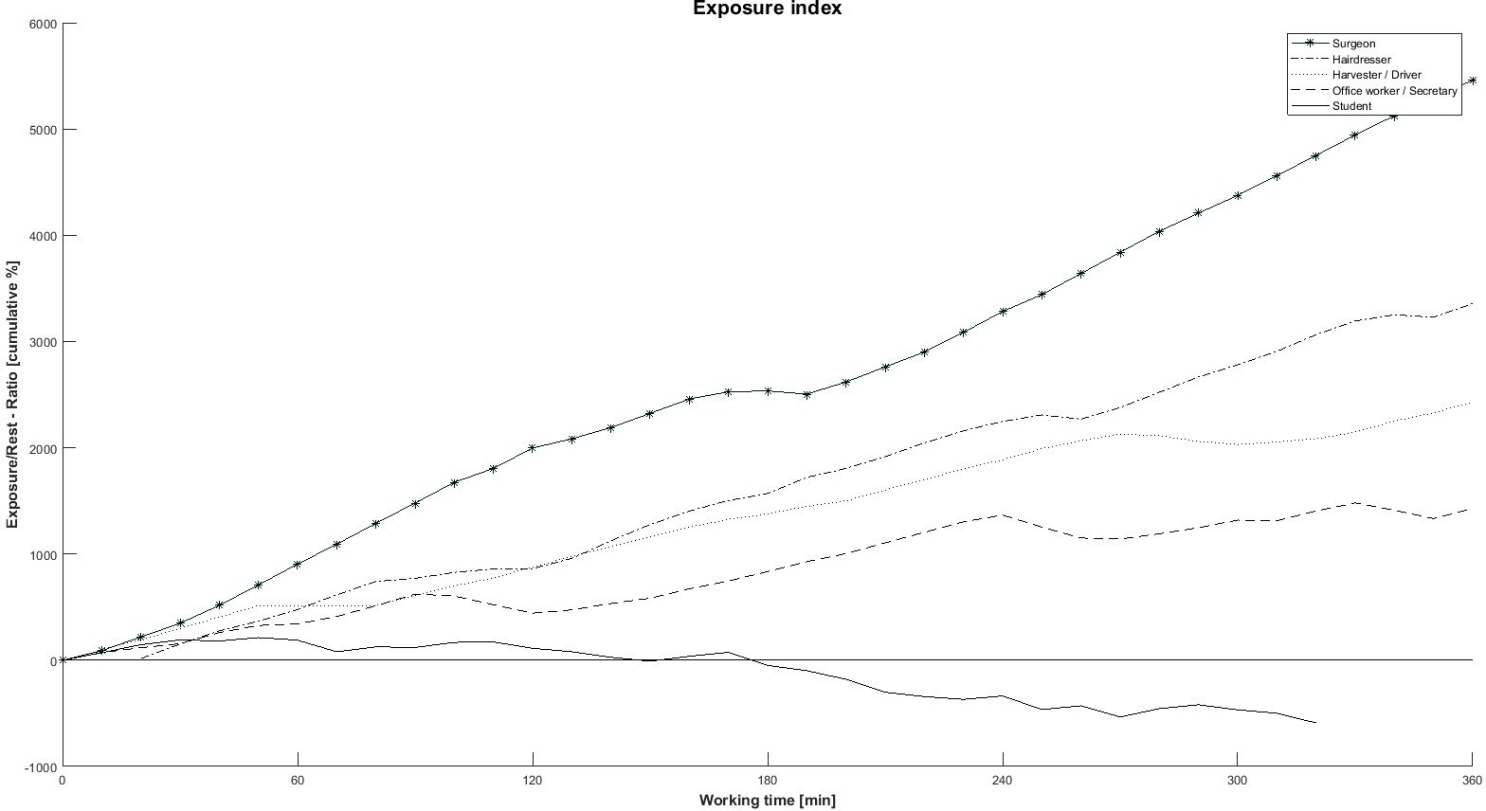
Calculated cumulative exposure/rest ratio of five participants from different professions.

Fig 1 presents an example of the calculated EI for the left trapezius muscle during working hours, featuring data from five participants in various occupations. The figure visually demonstrates the variation in exposure levels not only across different professions but also within the course of a workday. Notably, in the case of the surgeon depicted in Fig 1, there is a consistent increase in the exposure-to-rest ratio, whereas the student appears to experience a “recovery” period in the trapezius muscle after 3 h of work.

The values for the EI at 2, 4, 6, and 8 h into the workday were employed for the subsequent analyses. It is worth noting that the number of participants included in the analysis diminishes as the working hours increase, owing to variations in measurement durations. To maximize participant inclusion, an individualized approach was adopted. Specifically, the slope of the EI (b_EI_) for both the left and right trapezius muscles was calculated via linear regression for each participant. The calculated slope of the EI can be seen as a theoretical average slope of the load in relation to the working time. A doubling of the working time would then mean a doubling of the load.

### Subjective measures–neck pain

The final dataset comprised subjective measures of neck pain and included various types of questions. To conduct a comprehensive data analysis, it was necessary to merge different pain intensity scales and temporal periods of pain occurrence to encompass all datasets. This amalgamation of diverse pain measurement types was executed systematically, resulting in seven steps of combined cross-sectional pain measures (Table 2) and four steps of combined longitudinal pain measures (Table 3). The authors decided to include at first measures with the most accurate scale (VAS), sorted after the most actual temporal occurrence (pain after work), followed by including the categorial scales.

**Table 2:** Number of included participants in various steps of pain variable combination–cross-sectional data.

Neck pain		Included	Dataset												
	Temporal occurrence	scale	[34]	[35]	[36]	[37]	[38]	[39]	[40–44]	[45, 46]	[50, 51]	[47]	[48]	[49]	Total
**Step 1**	Pain after work	VAS	21	96	18	60	43								**238**
**Step 2**	+ pain for the past 7 days	VAS	21	96	18	60	43	11	146	53					**448**
**Step 3**	+ pain for the past 4 weeks	VAS	21	96	18	60	43	11	146	231					**626**
**Step 4**	+ pain for the past 12 months	VAS	21	96	18	60	43	11	146	231	43				**669**
**Step 5**	+ pain after/before the working day	0 1 2	21	96	18	60	43	11	146	231	43	10			**679**
**Step 6**	+ pain for the past 4 weeks	0 1 2 3	21	96	18	60	43	11	146	231	43	10	42		**721**
**Step 7**	+ pain for the past 7 days	0 1	21	96	18	60	43	11	146	231	43	10	42	10	**731**

Specification of included pain scales:

VAS - Visual analog scale (0:10)

(0 1 2) - (0) no pain, (1) slightly bothered, (2) quite bothered

(0 1 2 3) - (0) no pain, (1) slight pain, (2) substantial pain, (3) very bothered

(0 1) - (0) no, (1) yes

**Table 3:** Number of included participants in various steps of pain variable combination–longitudinal data.

Neck pain		Included	Dataset						
	Temporal occurrence	scale	[34]	[38]	[37]	[44]	[48]	[35]	Total
**Step 1**	6 months	VAS	18	40					**58**
**Step 2**	+ 12 months	VAS	18	42	60				**120**
**Step 3**	+ 24 months	VAS	18	42	60	51			**171**
**Step 4**	+ 12 months	0 1 2 3	18	42	60	51	32	56	**259**

Specification of included pain scales:

VAS - Visual analog scale (0:10)

(0 1 2 3) - (0) no pain, (1) slight pain, (2) substantial pain, (3) very bothered

**Fig 2:**
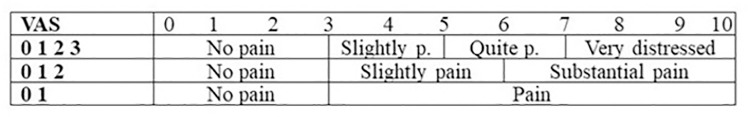
Classification of various pain scales compared to Visual Analog Scale.

Given the heightened sensitivity of the visual analog scale (VAS) in comparison to other scales, VAS was selected as the primary scale for all seven or four combined pain measures [60]. When including the categorical pain scales, they were then transformed into corresponding VAS values (Fig 2), specifically, they were substituted with values aligned with the lowest corresponding VAS value.

For all categorical scales, a value of zero was retained to represent “no pain.” In the case of the 4-point scale (ranging from “no pain” to “slightly painful” and beyond), the values were replaced with zero, three, five, and seven, respectively. Similarly, the 3-point scale (ranging from “no pain” to “substantial pain”) was substituted with the values zero, three, and six. The dichotomous scale indicating the presence or absence of pain (yes/no) was replaced with a value of three. For each ordinal scale, the specific values were replaced by the lowest corresponding value on the VAS. Example: Very distressed on the 4-point scale (0, 1, 2, 3) was replaced by VAS-value 7.

Since not every dataset contained longitudinal data, it must be mentioned that analyses of these data include a different composition of participants compared to the steps of neck pain variables in cross-sectional analyses. A detailed overview of the included professions depending on the steps of neck pain is given in S4 and S5 Tables.

### Statistical analyses

We conducted separate linear regression analyses to investigate the relationships between individual factors, (sex, age, height, weight, BMI, smoking status, and profession), and each different step of combined neck pain measurements, both cross-sectional and longitudinal. Factors significantly associated with neck pain (i.e., height and sex) were incorporated as confounders in subsequent adjusted linear regression. In these analyses, various EMG parameters (percentiles, gaps, SUMA, EI) were considered predictors of interest and assessed in relation to their effect on cross-sectional and longitudinal neck pain. The statistical analysis was performed using IBM SPSS Statistics 25, located in Armonk, New York, United States. The significance level was set at α ≤ 0.05.

### Sub-analyses

Furthermore, all analyses were conducted exclusively using EMG data from the dominant arm, but this was contingent on the availability of pertinent information from the participants.

### Ethics approval and consent to participate

The consent for the pooling, storing and analysis of the various anonymized datasets was given by the Norwegian Centre for Research Data. Because ethical approval was already obtained in the original studies of the pooled data, there is no need for further ethical approval by the Regional Committee for Medical Research Ethics (Act on Medical and Health Research §§2, section 1.4.d, Application title: “Muskelaktivering og pauser under arbeid og nakkesmerter”; application number: 275929; REK sør-øst B, Gullhaugveien 1–3, 0484 Oslo). Regarding GDPR, all data are anonymized, and it is not possible to identify any individuals.

The included data material of each study was anonymized accessed from 01.11.2021 to 30.06.2022. Written consent was obtained from all subjects involved in the included studies.

## Results

For all participants, the average duration of the recorded working shift was 6 h and 42 min (SD: 1 h and 55 min, Range: 60–692 min).

Significant associations between individual variables and neck pain measures were observed for height and sex, as indicated in steps 2 to 7 (Table 4). Smaller individuals and females reported higher scores for neck pain. Consequently, subsequent analyses were adjusted to account for height and sex.

**Table 4:** β-values for unadjusted regression analyses for general variables to neck pain variables (steps 1 to 7).

Neck pain:	Step 1	Step 2	Step 3	Step 4	Step 5	Step 6	Step 7
Age	-0.029	0.059	0.006	0.027	0.022	0.046	0.046
Weight	0.122	-0.074	-0.068	-0.06	-0.06	-0.034	-0.036
Height	0.048	**-0.137** **	**-0.103** *	**-0.092** *	**-0.091** *	**-0.081** *	**-0.079** *
BMI	-0.04	-0.035	-0.041	-0.038	-0.038	-0.031	-0.031
Sex	-0.108	**0.242** **	**0.162** **	**0.153** **	**0.149** **	**0.149** **	**0.14** **
Smoking	**-0.162** *	**-0.17** **	0.024	0.025	0.025	0.022	0.022
Snus	0.093	0.093	0.093	0.098	0.098	0.045	0.045

Gray shaded: negative associations. Significant results are in bold.

* p < 0.05

** p < 0.01

### Cross-sectional analyses

Regression analyses examining RRT, total duration with low muscular activity, gaps in short muscular rest, and percentiles in relation to neck pain revealed no significant associations for the left side of the body (Table 5). While the associations between neck pain and the 50th and 90th percentiles, as well as RRT, were predominantly negative, positive associations were noted with the duration of low muscular activity, the number of gaps, and the 10th percentile of muscle activity, although these results did not reach statistical significance.

**Table 5:** β-values for adjusted regression analysis for RRT, percentiles, gaps, SUMA-periods, and EI to cross-sectional neck pain variables (steps 1 to 7) ^#^.

Neck pain:	Step 1	Step 2	Step 3	Step 4	Step 5	Step 6	Step 7
**Left**			**				
RRT (<0.05% MVE)	0.001	0.024	0.026	0.023	0.023	0.008	0.009
Duration 2–10% MVE	-0.022	0.053	0.042	0.061	0.062	0.064	0.064
Nr Gaps/min <0.5%	0.104	0.020	0.043	0.031	0.033	0.032	0.033
10th percentile	0.042	-0.048	-0.051	-0.054	-0.024	-0.020	-0.020
50th percentile	-0.014	0.012	0.006	-0.004	-0.011	-0.005	-0.005
90th percentile	0.004	-0.035	-0.037	-0.040	-0.052	-0.042	-0.044
***SUMA-periods*:**							
1.5s - 5s	**0.149** *	**0.112** *	**0.13** **	**0.111** *	**0.112** *	**0.109** *	**0.109** *
5s - 10s	0.110	**0.122** *	**0.134** **	**0.120** *	**0.122** *	**0.114** *	**0.113** *
10s - 20s	0.075	**0.135** *	**0.143** **	**0.141** **	**0.143** **	**0.128** **	**0.127** **
20s - 60s	-0.025	**0.103** *	**0.102**	**0.109** *	**0.110** *	**0.091** *	**0.089** *
1min - 2min	**-0.138** *	-0.003	-0.014	-0.005	-0.004	-0.006	-0.006
2min - 4min	-0.114	-0.015	-0.023	-0.025	-0.022	-0.019	-0.021
4min - 8min	-0.070	-0.038	-0.047	-0.044	-0.044	-0.035	-0.038
8min - 10min	-0.035	-0.048	-0.053	-0.061	-0.060	-0.054	-0.049
10min - 20min	0.009	**-0.099** *	**-0.103** *	**-0.101** *	**-0.107** *	**-0.066** *	-0.068
>20min	-0.017	**-0.121** *	**-0.123** *	**-0.112** *	**-0.101** *	**-0.094** *	-0.085
***Exposure Index*:**							
Slope	-0.003	-0.017	-0.019	-0.010	-0.013	-0.001	-0.003
2 h	0.009	-0.016	-0.019	-0.011	-0.015	-0.007	-0.008
4 h	0.013	-0.031	-0.032	-0.029	-0.029	-0.017	-0.020
6 h	-0.004	-0.040	-0.043	-0.049	-0.043	-0.019	-0.022
8 h	0.015	0.006	0.006	0.006	0.006	0.010	0.010
**Right**							
RRT (<0.05% MVE)	0.041	0.055	-0.049	-0.047	-0.043	-0.045	-0.045
Duration 2–10% MVE	0.007	0.044	-0.001	0.015	0.02	0.021	0.02
Nr Gaps/min <0.5%	0.065	0.067	0.04	0.037	0.036	0.044	0.043
10th percentile	0.027	-0.057	0.053	0.041	0.038	0.04	0.042
50th percentile	-0.043	-0.069	**0.092** *	**0.079** *	0.068	0.061	0.061
90th percentile	-0.093	**-0.108** **	0.06	0.054	0.049	0.046	0.046
***SUMA-periods*:**							
1.5s - 5s	0.102	**0.102** *	0.053	0.048	0.052	0.06	0.06
5s - 10s	0.087	**0.124** *	0.068	0.061	0.067	0.073	0.072
10s - 20s	0.025	**0.125** *	0.07	0.069	0.076	**0.077** *	**0.077** *
20s - 60s	-0.065	0.093	0.055	0.06	0.067	0.065	0.065
1min - 2min	**-0.138** *	0.042	0.021	0.034	0.038	0.03	0.027
2min - 4min	-0.122	-0.027	0.021	0.028	0.032	0.024	0.018
4min - 8min	-0.037	-0.055	0.012	0.013	0.007	0.01	0.008
8min - 10min	0.01	-0.089	-0.051	-0.058	-0.056	-0.054	-0.054
10min - 20min	-0.043	**-0.162** *	-0.061	-0.064	-0.061	-0.059	-0.058
>20min	0.117	-0.067	-0.01	-0.019	-0.038	-0.037	-0.032
***Exposure Index*:**							
Slope	-0.051	-0.063	0.055	0.059	0.052	0.049	0.049
2 h	-0.059	-0.081	0.054	0.059	0.053	0.047	-0.068
4 h	-0.083	-0.087	0.070	0.070	0.066	0.063	-0.073
6 h	-0.118	-0.112	0.068	0.063	0.058	0.063	-0.091
8 h	-0.316	-0.238	0.135	0.135	0.135	0.142	-0.212

^#^ Adjusted for height and sex. Gray shaded: negative associations. Significant results are in bold.

* p < 0,05

** p < 0,01.

Conversely, for the right side of the body, somewhat contrasting associations were observed (Table 5). Significant associations were found for the 50th percentile (positive associations with neck pain in steps 3 and 4) and the 90th percentile (negative association with neck pain in step 2) of the right trapezius muscle activity. In the case of the right side of the body and neck pain in step 2, participants with higher pain levels exhibited lower peak loads.

Although most participants were right-handed, additional analyses pertaining to the dominant side of the body corroborated the findings from the left side (S2 Table).

Significant positive associations between neck pain and SUMA in the left trapezius were observed for shorter periods (up to 60 s, from step 2 to step 7) and longer periods (> 10 min, from step 2 to step 6). Similar but less significant results were found for the right trapezius. Additional analyses conducted on the dominant side of the body yielded similar outcomes. For shorter periods, the associations were predominantly positive, whereas longer periods showed negative associations (with the exception of SUMA–periods lasting 8 to 10 min; S2 Table). Workers with a higher number of shorter periods of muscle activity tended to report higher pain scores. Conversely, lower pain levels were reported when working with a greater number of longer periods of SUMA.

In the cross-sectional analysis of the left body site (Table 5), participants with higher pain scores generally exhibited a lower EI within the first 6 h of work. However, for the right trapezius muscle, the results varied depending on the specific neck pain variables and, consequently, the professions included in the analyses. Negative associations were observed for steps 1, 2, and 7, whereas associations were positive for neck pain in steps 3 to 6. When conducting analyses with the dominant body side, negative associations were evident for neck pain in steps 1, 2, and 6, whereas associations for steps 4, 5, and 7 were predominantly positive (S2 Table).

### Longitudinal analyses

In our longitudinal analysis of neck pain variables, we identified significant positive associations between the 90th percentile of left trapezius muscle activity and the 10th, 50th percentile, and SUMA-periods exceeding 8 min for right trapezius muscle activity (Table 6).

**Table 6:** β-values for adjusted regression analysis for RRT, percentiles, gaps, SUMA-periods, and EI to longitudinal neck pain variables (steps 1 to 4) ^#^.

Neck pain:	Step 1	Step 2	Step 3	Step 4
**Left**				
RRT (<0.05% MVE)	-0.231	-0.053	-0.031	-0.046
Duration 2–10% MVE	0.059	0.003	0.052	-0.001
Nr Gaps/min >0.5%	-0.044	0.036	-0.048	-0.032
10th perc	0.251	0.083	0.050	0.064
50th perc	0.269	0.079	0.038	0.030
90th perc	**0.305** *	0.048	-0.001	0.012
***SUMA-periods*:**				
1.5s - 5s	-0.184	0.089	0.074	0.022
5s - 10s	-0.232	0.089	0.072	0.002
10s - 20s	-0.109	0.068	0.060	-0.036
20s - 60s	0.011	-0.007	0.009	-0.062
1min - 2min	-0.043	-0.143	-0.079	-0.049
2min - 4min	0.069	-0.129	-0.054	0.039
4min - 8min	0.163	0.012	0.053	0.114
8min - 10min	0.272	0.129	0.102	0.067
10min - 20min	0.192	0.132	0.033	0.087
>20min	0.277	0.098	0.005	-0.016
***Exposure Index*:**				
Slope	0.265	0.061	0.040	0.053
2 h	**0.333** *	0.103	0.078	0.049
4 h	0.319	0.059	0.028	0.042
6 h	**0.452** *	0.035	-0.003	0.075
8 h	0.385	-0.135	-0.165	-0.160
**Right**				
RRT (<0.05% MVE)	-0.202	-0.072	-0.031	-0.057
Duration 2–10% MVE	0.021	0.050	0.098	0.093
Nr Gaps/min >0.5%	-0.151	-0.054	-0.033	-0.041
10th perc	**0.304** *	0.155	0.051	0.082
50th perc	**0.339** *	0.169	0.032	0.010
90th perc	0.181	0.065	-0.020	-0.022
***SUMA-periods*:**				
1.5s - 5s	-0.243	-0.132	-0.068	-0.075
5s - 10s	-0.253	-0.125	-0.050	-0.067
10s - 20s	-0.210	-0.078	-0.017	-0.081
20s - 60s	-0.142	-0.019	0.054	-0.043
1min - 2min	-0.036	0.073	0.131	0.023
2min - 4min	0.131	0.146	0.104	0.055
4min - 8min	0.232	0.113	0.046	0.064
8min - 10min	**0.303** *	0.033	-0.133	-0.024
10min - 20min	**0.365** *	0.153	-0.075	-0.026
>20min	**0.363** *	0.017	-0.045	0.082
***Exposure Index*:**				
Slope	0.274	0.101	0.037	0.048
2 h	**0.310** *	0.141	0.068	0.048
4 h	0.301	0.083	0.024	0.036
6 h	**0.462** *	0.020	-0.026	0.062
8 h	0.297	-0.203	-0.233	-0.228

^#^ Adjusted for height and sex.

Gray shaded: negative associations.

Significant results are in bold.

* p < 0.05

** p < 0.01

While examining the associations with the RRT on both sides of the body (steps 1 to 4), we consistently observed negative correlations; however, these correlations were not statistically significant. A discernible pattern was not readily apparent.

It is worth noting that on the right side of the body, supported by the findings from analyses involving the dominant body side, we observed results that contradicted those from cross-sectional analyses. Specifically, when it came to SUMA-periods, we found that shorter periods (less than 2 min) exhibited negative associations, while longer periods (greater than 2 min) displayed positive associations.

In our longitudinal analysis of the EI, we observed associations that were contrary to those found in cross-sectional results (Table 6). Specifically, individuals with a higher exposure level consistently reported higher pain scores. This trend was further substantiated through our analyses of the dominant body side (S3 Table).

### Objective ranking of professions according to cumulative exposure

Significant differences in mean ranks among various professions were observed at all time points (p < 0.001). Table 7 displays the ranked occupations based on the EI after two and six working hours.

**Table 7:** Mean ranks for EI of right trapezius muscle after two and six working hours.

T 2h	n	Mean Rank	T 6h	n	Mean Rank
Windscreen inspection	9	203.89	Student	3	42.33
Student	5	215.60	Rubber mixing	1	115.00
Office worker / Secretary	104	223.31	Firefighter	1	146.00
Foreman	5	253.20	Working with various tasks	5	155.80
Working with various tasks	7	254.29	Office worker / Secretary	50	159.18
Assistant worker	3	264.67	Foreman	5	176.80
Bricklayer	22	267.41	Mechanic	4	183.75
Harvester / Driver	93	267.81	Bricklayer	19	196.95
Mechanic	4	283.75	Harvester / Driver	75	211.04
Concrete worker	37	317.11	Assistant worker	3	213.33
Rubber mixing	8	327.50	Concrete worker	32	225.00
Other occupations	14	331.14	Other occupations	14	229.93
Carpenter	15	348.93	Engineer	3	237.33
Engineer	3	350.00	Electrician	12	249.17
Cook or kitchen helper	8	353.50	Cook or kitchen helper	7	252.57
Firefighter	2	369.50	Health care personal	52	266.35
Meat cutter	35	374.74	Meat cutter	27	273.26
Electrician	16	390.31	Retail personal	36	273.53
Health care personal	73	391.74	Machine operator	4	277.75
Machine operator	4	402.50	Carpenter	11	282.09
Hairdresser	36	407.50	Warehouse worker	35	304.00
Retail personal	45	417.47	Hairdresser	31	304.77
Postal worker	36	434.97	Postal worker	33	342.33
Warehouse worker	38	437.63	Cleaner	11	361.09
Assembly worker	25	443.32	Assembly worker	23	366.87
Project manager/leader	12	473.75	Project manager/leader	9	370.89
Cleaner	12	496.17	Gardener/forest worker	4	421.00
Helicopter pilot	2	530.00	Brewery worker	4	433.00
Surgeon	19	569.79	Surgeon	6	463.00
Brewery worker	4	592.75			
Gardener/forest worker	5	603.60			
**Total**	**701**	** **	**Total**	**520**	** **

## Discussion

To investigate the relationship between trapezius muscle activity during working hours and neck pain, this study utilized several datasets obtained from seven Scandinavian research institutes. In contrast to previous research, our objective was to scrutinize the frequency of breaks, the duration of muscular rest, and SUMA while incorporating a larger number of participants from diverse occupational backgrounds. Despite our initial goal of collecting data from a total of 1500 participants, we ultimately included 731 individuals. Nevertheless, the authors maintain that this participant count, coupled with the high-quality EMG measurements used to characterize trapezius muscle work exposure, represents an advantage compared to previous studies.

The results revealed distinct associations between height and sex with measures of neck pain, whereas other individual factors such as age, weight, smoking, snus usage, and BMI, as well as one’s profession, did not show significant associations. Individuals of smaller stature and females reported higher levels of pain on the scale. This observation may be attributed to the fact that smaller body sizes could require more lifting of the arms for the same work tasks, thereby increasing the workload and leading to neck pain. The higher reported pain among women aligns with findings from previous studies [61].

Regarding the RRT, duration of activity between 2 and 10% MVE, gap frequency and muscle activity levels (amplitude probability distribution function), almost no significant associations were identified either in cross-sectional or longitudinal analyses. However, concerning the right trapezius muscle, cross-sectional analyses revealed significant positive associations for the 50th percentile (neck pain steps 3 and 4) and negative associations for the 90th percentile (step 2), but these may be “accidental” findings. For the 50^th^ and 90^th^ percentiles, the additional analyses, including only data from the dominant arm, confirmed the results of the left body site. One possible explanation for this correlation could be that due to the wide range of occupational groups included, the left arm is used to support right-handed people in manual occupations. Since the 50th and 90th percentiles are the median and peak loads, this could support this hypothesis.

An issue for a long time has been about people with neck pain tensing their neck muscles more than painless people. There have been hypotheses that pain in a muscle has resulted in increased activity in supporting muscles for "relief", e.g. the pain-adaption model proposed by Lund [62]. This cross-sectional information has been used for diagnostic purposes, but we cannot say if the muscle activation pattern is a possible cause of neck pain. In a preventive context, it is most of interest if such a generally increased muscle activity can be a risk factor (or mediator, i.e., a possible explanatory mechanism) for later neck pain. Cross-sectional studies and laboratory studies have not found a consistent correlation [5, 13, 14, 63–68]. Generally, increased muscle activity does not appear to be related to concomitant pain. However, several findings are suggesting that people with neck pain tense the muscles more than pain-free by ’stress exposure’ [18, 69], which has also been seen in a prospective study before people developed neck pain [19].

The most noteworthy findings in this study pertain to the SUMA periods. In the case of the left trapezius muscle, numerous significant results emerged for SUMA-periods lasting up to 60 s (positive associations) and exceeding 10 min (negative associations) in cross-sectional analyses. Additionally, negative associations were observed for periods exceeding 1 min, although these did not reach statistical significance. Both the right trapezius and the analyses involving the dominant side of the body exhibited a similar pattern, albeit with slight shifts in the transition from positive to negative associations based on the duration of the SUMA-periods. These results may be in accordance with the previously mentioned pain-adaption model, which points to a protective muscle activity pattern when having pain [62]. Based on these results, it appears that individuals with higher levels of neck pain tend to work more frequently with shorter periods of SUMA in the short term while avoiding longer periods. One possible explanation for these findings could be that workers experiencing greater pain need to introduce more frequent short breaks to cope with their exposure, thereby avoiding prolonged periods of muscle strain.

Conversely, in longitudinal analyses, these patterns were reversed for both the right and left trapezius muscles, as well as the dominant arm. An overwhelming number of beta-values are negative for the short SUMA periods and positive for the longer periods. Significant findings were only observed for the right trapezius in SUMA-periods exceeding 8 min (associated with neck pain step 1, exhibiting positive associations). Data building on neck pain step 1 includes results from our lab, where we previously found a longitudinal relationship between long SUMA periods and neck pain [33, 70].

Interestingly, these results contradict those from one of the studies included in our analysis. In the cross-sectional analysis by Østensvik and colleagues, opposite associations were found for short and long periods of SUMA in the trapezius among harvesters, forwarders, and researchers [31, 38]. This discrepancy may be explained by the nature of the work tasks for harvesters and forwarders, which often involve fixed postures and limited opportunities for modifying their exposure, such as working seated in a cabin.

However, in the long term, the evidence suggests that working more frequently with longer periods of SUMA, without allowing for adequate muscle recovery or rest, may contribute to higher levels of pain. Although these results do not reach statistical significance, they align with findings from other studies included in our analysis, where a higher risk of neck pain was associated with working in sustained muscle activity periods exceeding 4 min [32, 33] or 8 min [31], and a lower risk was associated with more frequent shorter SUMA-periods [31].

It is worth noting that the inclusion of a wide range of occupations in this study may have attenuated the significance of the associations observed in the longitudinal analyses.

The purpose behind calculating the EI was to provide a descriptive and visual representation of exposure development throughout the working day. This index amalgamated and related the proportions of muscular rest, low muscular activity, and high muscular activity. For the left trapezius muscle, as well as in the analyses involving the dominant arm, the EI predominantly exhibited negative associations with neck pain. However, in longitudinal analyses, the associations were mostly positive.

The EI can be regarded as a comprehensive reflection of muscular loads, encompassing both exposure and recovery throughout the workday. It aligns with our findings concerning SUMA, suggesting that participants experiencing neck pain may exercise more caution in the short term, while prolonged and higher levels of exposure over time tend to result in pain.

As previously mentioned, heightened trapezius muscle activity can stem from both physical and psychosocial factors. For instance, tasks involving the use of hands above shoulder level have been associated with increased trapezius muscle activity [71]. However, it is worth noting that such work has also been identified as a strong predictor of neck pain in a prior review conducted by Mayer and colleagues [72]. In a preventive context, the use of arm support in various work tasks [73] or the adoption of exoskeletons [74] has the potential to reduce trapezius muscle activity.

The presence of variations in the EI among the included occupations, alongside the absence of significant associations between occupation and neck pain, lends support to the notion that muscle activity alone may not be the sole cause of neck pain [75]. It underscores the importance of considering psychological and social factors as potential moderators in the analyses [50, 76, 77].

We also aimed to incorporate psychological factors into our analyses. However, upon reviewing the available data on psychological and social factors across the different datasets, we encountered significant variability and incomplete information, rendering this endeavor unfeasible.

As mentioned above, the inclusion of a wide range of occupations may have attenuated the significance of the associations, not only in longitudinal, but also in cross-sectional analyses. Further examinations of sedentary and non-sedentary occupations or various job categories depending on the degree of physical strain will probably be discussed in a subsequent paper.

### Methodological considerations

For this study, electromyography (EMG) data from the upper trapezius muscle were collected bilaterally throughout a typical workday by consolidating data from multiple studies. Nevertheless, despite the benefits of having a substantial dataset, several challenges needed to be addressed.

(1) In general, all of the EMG data included in this study were measured on a single workday. Consequently, it is reasonable to assume that these measurements may not accurately reflect typical or commonplace work tasks, which can vary over time. For instance, office or assembly line workers often engage in repetitive and monotonous tasks, whereas construction workers likely encounter a wide range of tasks throughout a project or year.

(2) All EMG datasets underwent processing in accordance with standardized guidelines. However, variations in time for data collection, procedures for MVE, filtering, and RMS formatting were noted. Each individual measurement underwent a quality check by the responsible author, followed by conversion to a standardized RMS format and post-processing with a common noise-canceling correction. Consequently, an attempt was made to establish a consistent level of quality across the final dataset for subsequent analyses. Unfortunately, it was not feasible to obtain all individual measurements in their raw data format, which would have allowed for uniform processing from the outset. Nevertheless, given the adherence to standards in the respective studies and the quality assurance steps mentioned, the authors maintain confidence in the suitability of the pooled dataset for generating scientifically meaningful results.

(3) The authors acknowledge that there are various approaches to combining the different scales of the back pain questionnaires, and this decision was made through a collaborative consensus of all authors. Deliberately, the VAS was selected as the ultimate scale, ranging from step 1 to step 7 for cross-sectional analysis and step 4 for longitudinal neck pain analysis. This choice aimed to minimize any loss in scale quality. However, it is essential to recognize that, despite the increasing number of participants, as one progresses to higher steps of combined neck pain measures, the scale’s accuracy tends to diminish. Hence, this factor should be taken into account when interpreting the results. Nonetheless, it is noteworthy that the results at higher steps often exhibit similar trends to those at lower steps.

(4) As previously discussed in the introduction, muscle activity in the neck is influenced by both physical and psychosocial factors. Job demands, job control, and workplace support have been identified as potential risk factors that could impact the relationship between muscle activity and neck pain. While some of the included datasets did contain information regarding psychosocial exposures, the diversity of this data made it impractical to combine different types of measures or scales. For future supplementary analyses focusing on subgroups within the pooled dataset, incorporating these measures into the analysis may be a viable option.

### Conclusion

This study afforded the authors the opportunity to simultaneously examine the relationship between trapezius muscle activity during the workday and neck pain across a large number of participants representing a diverse range of occupations. In the short term, neck pain appears to lead to shorter periods of trapezius muscle activity and avoidance of sustained activity over longer durations. Conversely, in the long term, prolonged periods of SUMA with fewer breaks appear to be predictive of neck pain. Notably, occupation alone does not appear to be a predictive factor for neck pain despite variations in the overall muscular load observed. To enhance predictive accuracy, the incorporation of additional factors, such as psychosocial or organizational variables, is necessary.

## Supporting information

S1 Table Included professions by dataset. The table shows the number of employees in each occupation included in each specific dataset.
(PDF)

S2 Table β-values for adjusted regression analysis for RRT, percentiles, gaps, SUMA-periods, and EI to cross-sectional neck pain variables (step 1 to 7) #—analysis on dominant arm. # Adjusted for height and sex. Significant results in bold. * p < 0,05, ** p < 0,01. Gray shaded: negative associations. For step 3 no more participants were included for analyses compared to step 2. Therefore, the β-values for step 3 have been removed from the table.
(PDF)

S3 Table β-values for adjusted regression analysis for RRT, percentiles, gaps, SUMA-periods, and EI to longitudinal neck pain variables (step 1 to 4) #—analyses on dominant arm. # Adjusted for height and sex. Significant results in bold. * p < 0,05, ** p < 0,01. Gray shaded: negative associations. For step 3 no more participants were included for analyses compared to step 2. Therefore, the β-values for step 3 have been removed from the table.
(PDF)

S4 Table Included professions in cross-sectional analyses depending on neck pain variable. The table shows the number of employees in each occupation included in each specific step of neck pain.
(PDF)

S5 Table Included professions in longitudinal analyses depending on neck pain variable. The table shows the number of employees in each occupation included in each specific step of neck pain.
(PDF)

S1 Dataset (SAV)
