# Supplementary material for: Frequency of breaks, amount of muscular rest, and sustained muscle activity related to neck pain in a pooled dataset
Source: PLoS One. 2024 Jun 25;19(6):e0297859. doi: 10.1371/journal.pone.0297859 (PMC11198897; doi:10.1371/journal.pone.0297859)
Supplement: S1 Table — The table shows the number of employees in each occupation included in each specific dataset. (PDF) [file pone.0297859.s001.pdf]

Profession	Reference / dataset												Total
	[34]	[35]	[36]	[37]	[38]	[39]	[40-44]	[45, 46]	[50, 51]	[47]	[48]	[49]	
Assembly worker								14	11				25
Assistant worker		2						1					3
Brewery worker								4					4
Bricklayer		3						19					22
Carpenter		17											17
Cleaner		2										10	12
Concrete worker		7						30					37
Cook or kitchen helper		8											8
Electrician											16		16
Engineer		3											3
Firefighter								2					2
Foreman		5											5
Gardener / forest worker								5					5
Hairdresser	21										15		36
Harvester / driver				60	25			9					94
Health care personal		36					40						76
Helicopter pilot			18										18
Machine operator								4					4
Meat cutter								27	8				35
Mechanic								4					4
Office worker / secretary					18		79	8	2				107
Postal worker								36					36
Project manager / leader		8						5					13
Retail personal							27	18					45
Rubber mixing									8				8
Student											5		5
Surgeon		1				11				10			22
Warehouse worker								35	3				38
Windscreen inspection									10				10
Other occupations		1						7			6		14
Working with various tasks		3						3	1				7
**Total**	**21**	**96**	**18**	**60**	**43**	**11**	**146**	**231**	**43**	**10**	**42**	**10**	**731**
