# Supplementary material for: Frequency of breaks, amount of muscular rest, and sustained muscle activity related to neck pain in a pooled dataset
Source: PLoS One. 2024 Jun 25;19(6):e0297859. doi: 10.1371/journal.pone.0297859 (PMC11198897; doi:10.1371/journal.pone.0297859)
Supplement: S2 Table — # Adjusted for height and sex. Significant results in bold. * p < 0,05, ** p < 0,01. Gray shaded: negative associations. For step 3 no more participants were included for analyses compared to step 2. Therefore, the β-values for step 3 have been removed from the table. (PDF) [file pone.0297859.s002.pdf]

| **Pain:** | **Step 1** | **Step 2** | **Step 3** | **Step 4** | **Step 5** | **Step 6** | **Step 7** |
|---|---|---|---|---|---|---|---|
| RRT | 0,036 | 0,007 | | -0,008 | -0,015 | -0,012 | -0,016 |
| Duration 2 - 10 MVE | -0,013 | 0,029 | | 0,046 | 0,060 | 0,043 | 0,036 |
| L Nr Gaps/min >0.5% | 0,120 | 0,099 | | 0,076 | 0,071 | 0,061 | 0,053 |
| Perc 10th | 0,022 | 0,036 | | 0,032 | 0,037 | 0,022 | 0,036 |
| Perc 50th | -0,047 | -0,038 | | -0,031 | -0,041 | -0,035 | -0,011 |
| Perc 90th | -0,069 | -0,080 | | -0,071 | -0,080 | -0,062 | -0,040 |
| ***SUMA – periods:*** | | | | | | | |
| 1.5s - 5s | **0,177*** | **0,151*** | | 0,118 | 0,112 | 0,101 | 0,097 |
| 5s - 10s | 0,134 | 0,116 | | 0,099 | 0,092 | 0,075 | 0,070 |
| 10s - 20s | 0,078 | 0,065 | | 0,077 | 0,072 | 0,046 | 0,043 |
| 20s - 60s | -0,027 | -0,026 | | 0,001 | -0,005 | -0,030 | -0,039 |
| 1min - 2min | **-0,133*** | -0,120 | | -0,094 | -0,098 | -0,088 | -0,100 |
| 2min - 4min | -0,100 | -0,075 | | -0,076 | -0,073 | -0,056 | -0,069 |
| 4min - 8min | -0,078 | -0,060 | | -0,046 | -0,049 | -0,024 | -0,027 |
| 8min - 10min | -0,042 | 0,013 | | 0,003 | 0,007 | 0,011 | 0,019 |
| 10min - 20min | -0,036 | -0,019 | | -0,026 | -0,005 | 0,035 | 0,034 |
| >20min | -0,058 | -0,051 | | -0,041 | -0,053 | -0,061 | -0,031 |
| ***Exposure Index:*** | | | | | | | |
| Slope | -0,035 | -0,010 | | 0,007 | 0,012 | 0,010 | 0,018 |
| 2h | -0,011 | -0,013 | | 0,003 | 0,009 | -0,007 | 0,002 |
| 4h | -0,021 | -0,021 | | -0,015 | 0,006 | -0,004 | 0,004 |
| 6h | -0,052 | -0,052 | | -0,070 | -0,037 | -0,003 | 0,010 |
| 8h | -0,074 | -0,074 | | -0,074 | -0,074 | -0,063 | -0,063 |
