# Supplementary material for: Frequency of breaks, amount of muscular rest, and sustained muscle activity related to neck pain in a pooled dataset
Source: PLoS One. 2024 Jun 25;19(6):e0297859. doi: 10.1371/journal.pone.0297859 (PMC11198897; doi:10.1371/journal.pone.0297859)
Supplement: S3 Table — # Adjusted for height and sex. Significant results in bold. * p < 0,05, ** p < 0,01. Gray shaded: negative associations. For step 3 no more participants were included for analyses compared to step 2. Therefore, the β-values for step 3 have been removed from the table. (PDF) [file pone.0297859.s003.pdf]

**Pain:**	**Step 1**	**Step 2**	**Step 3**	**Step 4**
RRT	-0,193	-0,058		-0,039
Duration 2 - 10 MVE	0,087	0,053		-0,026
Nr Gaps 0.5 / min	0,022	0,043		0,016
Perc 10th	0,184	0,089		0,077
Perc 50th	0,197	0,079		0,014
Perc 90th	0,279	0,027		0,013
SUMA - periods				
1.5s - 5s	-0,133	-0,016		-0,035
5s - 10s	-0,192	-0,050		-0,072
10s - 20s	-0,079	-0,017		-0,102
20s - 60s	-0,005	-0,024		-0,103
1min - 2min	-0,050	-0,036		-0,043
2min - 4min	0,049	0,059		0,073
4min - 8min	0,054	0,032		0,112
8min - 10min	0,191	0,047		0,048
10min - 20min	0,194	0,112		0,089
>20min	0,296	0,067		0,048
Exposure Index:				
Slope	0,218	0,063		0,044
2h	0,277	0,104		0,027
4h	0,284	0,050		0,027
6h	0,489	0,044		0,110
8h	0,379	-0,240		-0,228
