# Supplementary material for: Frequency of breaks, amount of muscular rest, and sustained muscle activity related to neck pain in a pooled dataset
Source: PLoS One. 2024 Jun 25;19(6):e0297859. doi: 10.1371/journal.pone.0297859 (PMC11198897; doi:10.1371/journal.pone.0297859)
Supplement: S4 Table — The table shows the number of employees in each occupation included in each specific step of neck pain. (PDF) [file pone.0297859.s004.pdf]

Profession	Cross-sectional neck pain Step 1	Step 2	Step 3	Step 4	Step 5	Step 6	Step 7
Assembly worker			14	25	25	25	25
Assistant worker	2	2	3	3	3	3	3
Brewery worker			4	4	4	4	4
Bricklayer	3	22	22	22	22	22	22
Carpenter	17	17	17	17	17	17	17
Cleaner	2	2	2	2	2	2	12
Concrete worker	7	37	37	37	37	37	37
Cook or kitchen helper	8	8	8	8	8	8	8
Electrician						16	16
Engineer	3	3	3	3	3	3	3
Firefighter			2	2	2	2	2
Foreman	5	5	5	5	5	5	5
Gardener / forest worker			5	5	5	5	5
Hairdresser	21	21	21	21	21	36	36
Harvester / driver	85	85	94	94	94	94	94
Health care personal	36	76	76	76	76	76	76
Helicopter pilot	18	18	18	18	18	18	18
Machine operator			4	4	4	4	4
Meat cutter			27	35	35	35	35
Mechanic			4	4	4	4	4
Office worker / Secretary	18	97	105	107	107	107	107
Other occupations	1	5	8	8	8	14	14
Postal worker			36	36	36	36	36
Project manager / leader	8	8	13	13	13	13	13
Retail personal		27	45	45	45	45	45
Rubber mixing				8	8	8	8
Student						5	5
Surgeon	1	12	12	12	22	22	22
Warehouse worker			35	38	38	38	38
Windscreen inspection				10	10	10	10
Working with various tasks	3	3	6	7	7	7	7
**Total**	**238**	**448**	**626**	**669**	**679**	**721**	**731**
