# Supplementary material for: Frequency of breaks, amount of muscular rest, and sustained muscle activity related to neck pain in a pooled dataset
Source: PLoS One. 2024 Jun 25;19(6):e0297859. doi: 10.1371/journal.pone.0297859 (PMC11198897; doi:10.1371/journal.pone.0297859)
Supplement: S5 Table — The table shows the number of employees in each occupation included in each specific step of neck pain. (PDF) [file pone.0297859.s005.pdf]

Profession	Longitudinal neck pain Step 1	Step 2	Step 3	Step 4
Assistant worker				1
Bricklayer				1
Carpenter				10
Cleaner				2
Concrete worker				5
Cook or kitchen helper				2
Electrician				11
Engineer				2
Foreman				3
Hairdresser	18	18	18	29
Harvester / Driver	22	84	84	84
Health care personal			15	35
Office worker / Secretary	18	18	43	43
Other occupations				6
Project manager / leader				7
Retail personal			11	11
Student				4
Working with various tasks				3
**Total**	**58**	**120**	**171**	**259**
